# Sex influences clinical phenotype in frontotemporal dementia

**DOI:** 10.1007/s10072-022-06185-7

**Published:** 2022-06-08

**Authors:** Marta Pengo, Antonella Alberici, Ilenia Libri, Alberto Benussi, Yasmine Gadola, Nicholas J. Ashton, Henrik Zetterberg, Kaj Blennow, Barbara Borroni

**Affiliations:** 1 Department of Molecular and Translational Medicine, University of Brescia, Brescia, Italy; 2 Department of Clinical and Experimental Sciences, Neurology Unit, University of Brescia, Brescia, Italy; 3 Department of Neurological and Vision Sciences, Neurology Unit, ASST Spedali Civili, Brescia, Italy; 4 Institute of Neuroscience and Physiology, Department of Psychiatry and Neurochemistry, The Sahlgrenska Academy at the University of Gothenburg, Mӧlndal, Sweden; 5 Wallenberg Centre for Molecular and Translational Medicine, University of Gothenburg, Mӧlndal, Sweden; 6 Institute of Psychiatry, King’s College London, Maurice Wohl Clinical Neuroscience Institute, Psychology & Neuroscience, London, UK; 7 NIHR Biomedical Research Centre for Mental Health & Biomedical Research Unit for Dementia at South London and Maudsley NHS Foundation, London, UK; 8 Clinical Neurochemistry Laboratory, Sahlgrenska University Hospital, Mölndal, Sweden; 9 UK Dementia Research Institute at UCL, London, UK; 10 Department of Neurodegenerative Diseases, UCL Institute of Neurology, London, UK; 11 Hong Kong Center for Neurodegenerative Diseases, Hong Kong, China

**Keywords:** Frontotemporal dementia, Sex differences, Dementia, Gender

## Abstract

**Introduction:**

Frontotemporal dementia (FTD) encompasses a wide spectrum of genetic, clinical, and histological findings. Sex is emerging as a potential biological variable influencing FTD heterogeneity; however, only a few studies explored this issue with nonconclusive results.

**Objective:**

To estimate the role of sex in a single-center large cohort of FTD patients.

**Methods:**

Five hundred thirty-one FTD patients were consecutively enrolled. Demographic, clinical, and neuropsychological features, survival rate, and serum neurofilament light (NfL) concentration were determined and compared between sex.

**Results:**

The behavioral variant of FTD was more common in men, whereas primary progressive aphasia was overrepresented in women (*p* < 0.001). While global cognitive impairment was comparable, females had a more severe cognitive impairment, namely in Trail Making Test parts A and B (*p* = 0.003), semantic fluency (*p* = 0.03), Short Story Recall Test (*p* = 0.003), and the copy of Rey Complex Figure (*p* = 0.005). On the other hand, men exhibited more personality/behavioral symptoms (Frontal Behavior Inventory [FBI] AB, *p* = 0.003), displaying higher scores in positive FBI subscales (FBI B, *p* < 0.001). In particular, apathy (*p* = 0.02), irritability (*p* = 0.006), poor judgment (*p* = 0.033), aggressivity (*p* = 0.008), and hypersexuality (*p* = 0.006) were more common in men, after correction for disease severity. NfL concentration and survival were not statistically different between men and women (*p* = 0.167 and *p* = 0.645, respectively).

**Discussion:**

The present study demonstrated that sex is a potential factor in determining FTD phenotype, while it does not influence survival. Although the pathophysiological contribution of sex in neurodegeneration is not well characterized yet, our findings highlight its role as deserving biological variable in FTD.

**Supplementary Information:**

The online version contains supplementary material available at 10.1007/s10072-022-06185-7.

## Introduction

Sex influences on brain functioning have been gaining increasing attention in both basic and clinical sciences [1]. Differences between men and women have been demonstrated in physiological as well as in pathological conditions in the brain [2, 3], and the role of sex is emerging as a crucial biological variable in the field of neurodegenerative diseases [4, 5].

In the context of dementia, Alzheimer’s disease (AD) has been largely explored for sex dissimilarities, mainly prompted by the higher AD prevalence in women [6, 7]. A large body of research has unraveled different risk factors and hormone influence in AD, along with distinct clinical presentations and specific brain changes between males and females [6, 8–10].

Conversely, few studies are available about the role of sex in frontotemporal dementia (FTD), probably due to the similar disease prevalence in men and women in clinical cohort studies and the lack of epidemiological studies on a possible role of sex in this disease [11].

FTD is characterized by a wide heterogeneity in terms of clinical, genetic, and neuropathological features. Exploring the factors that might contribute to this heterogeneity may represent a great challenge. Different phenotypes have been described on the basis of presenting clinical symptoms: the behavioral variant of FTD (bvFTD), characterized by early behavioral and personality changes and executive dysfunction [12], and primary progressive aphasia (PPA), associated with progressive deficits in language [13]. In particular, the agrammatic variant of PPA (avPPA) presents with slow, effortful speech, and grammar deficits, whereas the semantic variant of PPA (svPPA) begins with difficulty finding words, particularly nouns, and single-word comprehension deficits.

A family history of dementia is found in 25–50% of the FTD patients with microtubule-associated protein tau (*MAPT)* and progranulin (*GRN*) mutations, and chromosome 9 open-reading-frame 72 (*C9orf72*) expansion as major pathogenetic determinants [14].

A few studies have suggested a link between sex and phenotypic presentation in FTD [15–17], and a higher female prevalence of *GRN* mutations in FTD has been reported [18]. However, these results are not conclusive, which prompted the present study, aimed at exploring sex influences on disease onset, cognitive and behavioral features, and survival and biological biomarkers of disease severity in a large, single-center cohort of FTD patients.

## Methods

### Participants

In the present study, patients fulfilling current clinical criteria for probable FTD [13, 19] were consecutively recruited at the Centre for Neurodegenerative Disorders, Department of Clinical and Experimental Sciences, University of Brescia, Italy, from July 2007 to July 2021.

All patients underwent a comprehensive evaluation of their past medical history, complete neurological examination, standardized neuropsychological assessment, and magnetic resonance imaging (MRI) of the brain.

In familial cases, based on the presence of at least one dementia case among first-degree relatives and early-onset sporadic cases, genetic screening for *GRN*, *C9orf72*, and *MAPT* was performed. Given the low frequency of *MAPT* mutations in Italy [20], we considered only the P301L mutation and we sequenced the entire *MAPT* gene only in selected cases.

In a subset of patients, cerebrospinal fluid analysis or PET amyloid was performed to exclude focal AD pathology, as previously reported [21]. FTD patients were followed over time and data on survival recorded.

### Neuropsychological and behavioral assessment

The standardized neuropsychological assessment included the Mini-Mental State Examination (MMSE), Trail-Making Test (part A and part B), letter and semantic fluencies, Token Test, Digit Span forward, Short Story Recall Test, and Rey Complex Figure (copy and recall) [22]. The level of functional independence was assessed with Basic Activities of Daily Living (BADL) [23].

Behavioral disturbances were rated by the Italian version of the Frontal Behavioral Inventory [24, 25]. Disease severity was measured by CDR Dementia Staging Instrument plus behavior and language domains from the National Alzheimer’s Coordinating Center and Frontotemporal lobar degeneration modules – sum of boxes (CDR plus NACC FTLD—SOB) [26].

### Neurofilament light (NfL) measurements

In a subgroup of patients (*n* = 188), the serum was collected to assess the concentrations of NfL. The serum was obtained by venipuncture, processed and stored in aliquots at − 80 °C according to standardized procedures. The serum NfL concentration was measured using a commercial NF-Light assay (Quanterix, Billerica, Massachusetts, USA) according to the manufacturer’s instructions. The lower limit of quantitation for serum NfL was 0.174 pg/ml. Measurements were carried out using a HD-X analyser (Quanterix, Billerica, Massachusetts, USA), and the operators were blinded to all clinical information. Quality control samples had mean intra- and inter-assay coefficients of variation of < 8% and < 20%, respectively.

### Statistical analysis

Continuous and categorical variables are reported as mean (± standard deviation) and *n* (%), respectively. Between-group differences in demographic and global neuropsychological measures were assessed using independent sample *t* test and chi-square’s test for continuous and categorical variables. Separate analyses of covariances (ANCOVAs) were conducted for each cognitive test and behavioral subitems, covarying for disease severity, assessed by CDR plus NACC FTLD – SOB. Differences in serum NfL were assessed with a one-way analysis of covariance (ANCOVA), corrected for age and disease severity. For comparisons of each cognitive test and FBI subitems, a correction for multiple comparisons was performed using the Benjamini–Hochberg False Discovery Rate (FDR).

Survival was calculated as the time from symptom onset to time of death or the last follow-up. A Kaplan–Meier survival analysis was conducted to compare overall survival between male and female patients. A *p*-value < 0.05 was considered significant. Data analyses were carried out using SPSS 21.0 software.

### Data availability

All study data, including study design, statistical analysis plan, and results, are available from the corresponding author, upon reasonable request.

## Results

### Participants

In the present study, 531 FTD patients were consecutively recruited, namely 345 patients with bvFTD [12], 118 with avPPA, and 68 with svPPA [13].

The study group consisted of 258 women (mean age 66.4 ± 8.1 years old) and 273 men (mean age 65.4 ± 8.4 years old). No significant differences in demographic characteristics were observed between groups, with the exception of the younger age at disease onset for male FTD patients (*p* = 0.047) (see Table 1).

**Table 1:** Demographic characteristics of FTD patients

Variable	All	Males	Females	*p-*values
Number	531	273	258	-
Age (years)	65.9 ± 8.3	65.4 ± 8.4	66.4 ± 8.1	0.147
Age at onset (years)	63.2 ± 8.2	62.5 ± 8.2	64.0 ± 8.2	**0.047**
Disease duration (years)	2.7 ± 2.3	2.8 ± 2.5	2.5 ± 1.9	0.102
Education (years)	9.1 ± 4.3	9.4 ± 4.4	8.7 ± 4.2	0.083
Pathogenetic mutation (%)	95 (18%)	44 (16%)	51 (20%)	0.168^
Phenotype, bvFTD vs PPA (%)	347 (65%)	201 (74%)	146 (57%)	** < 0.001^**

Demographic characteristics are expressed as mean ± standard deviation, unless otherwise specified

bvFTD = behavioral variant Frontotemporal Dementia; PPA = Primary Progressive Aphasia

*p-*values are determined by means of independent sample t-test comparison student t test, unless otherwise specified

^ *p-*values for Chi-Square test comparison

FTD-related pathogenic mutations were identified in 95 patients (*n* = 66 *GRN* mutations, *n* = 26 *C9orf72* expansions, *n* = 3 *MAPT* mutations). There were no sex differences within the prevalence of pathogenic mutations.

The main finding of our study is that the bvFTD phenotype was more common in men (74%), whereas PPA in women (57%, *p* < 0.001).

### Neuropsychological measures in female and male FTD patients

Men and women showed no differences in global disease severity, as measured with CDR plus NACC FTLD—SOB (see Table 2). Overall, men presented more frequently behavioral disturbances, whereas women had higher cognitive impairment when considering specific tasks.

**Table 2:** Clinical, behavioral and neuropsychological assessment of FTD patients

Variable	All	Males	Females	p-value
CDR plus NACC FTLD—SOB	6.8 ± 4.9	6.6 ± 4.7	7.1 ± 5.0	0.328°
MMSE	20.4 ± 12.8	20.7 ± 7.2	20.1 ± 16.8	0.620°
FBI A	12.2 ± 7.5	12.4 ± 7.5	12.0 ± 7.6	0.148^
FBI B	5.9 ± 5.8	6.8 ± 6.0	5.0 ± 5.4	** < 0.001^**
FBI AB	18.1 ± 11.7	19.1 ± 12.1	17.0 ± 11.3	**0.003^**
Trail Making Test, part A (sec)	125.4 ± 141.7	104.5 ± 125.1	150.4 ± 156.0	**0.003**^
Trail Making Test, part B (sec)	274.2 ± 156.1	245.7 ± 153.9	311.5 ± 151.7	**0.003**^
Fluency, letter	18.8 ± 11.1	19.3 ± 10.7	18.1 ± 11.5	0.452^
Fluency, semantic	23.6 ± 12.4	25.1 ± 12.6	22.0 ± 12.0	**0.03**^
Token Test	25.4 ± 8.2	26.7 ± 7.2	24.2 ± 9.0	0.173^
Digit Span forward	4.8 ± 1.4	4.9 ± 1.3	4.6 ± 1.5	0.142^
Short Story Recall Test	8.0 ± 4.9	9.0 ± 4.7	6.8 ± 4.8	**0.003**^
Rey Complex Figure, copy	23.8 ± 13.1	25.8 ± 14.6	21.7 ± 10.9	**0.005**^
Rey Complex Figure, recall	9.5 ± 7.7	9.8 ± 8.9	9.2 ± 6.2	0.452^

Behavioural and neuropsychological global measures are expressed as mean ± standard deviation

CDR plus NACC FTLD—SOB = Clinical Rating Scale plus National Alzheimer’s Coordinating Centre FTDL Sum of Boxes; MMSE = Mini Mental State Examination; FBI = Frontal Behavior Inventory

° *p-*values for independent sample t-test comparison student t test

^ *p-*values for one-way ANCOVA are expressed after adjusting for CDR plus NACC FTLD—SOB

All results are corrected for multiple comparisons (False Discovery Rate)

**Fig. 1:**
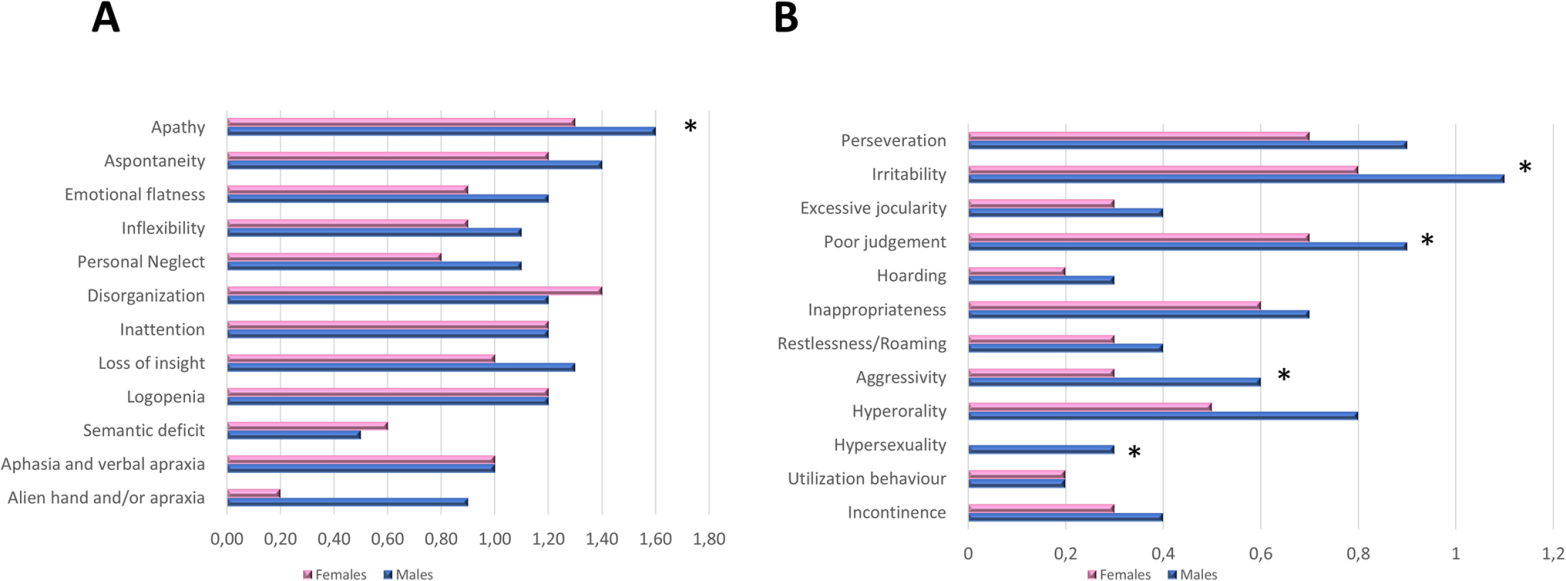
Frontal behavior inventory. **A** Bar graph reporting FBI subitems part A in male (blue) and female (pink) patients. **B** Bar graph reporting FBI subitems part B in male (blue) and female (pink) patients. ^*^Statistically different between males and females

We compared behavioral disturbances, assessed by the FBI scale, across the two groups. Men exhibited more personality/behavioral symptoms (FBI AB, *p* = 0.003), displaying higher scores in positive FBI subscales (FBI B, *p* < 0.001), with no differences in FBI A. In particular, looking at FBI subitems, men presented more severe apathy (1.6 ± 1.1 vs. 1.3 ± 1.1, *p* = 0.02), irritability (1.1 ± 1.0 vs. 0.8 ± 1.0, *p* = 0.006), poor judgment (0.9 ± 1.2 vs. 0.7 ± 1.0, *p* = 0.033), aggressivity (0.6 ± 0.9 vs. 0.3 ± 0.7, *p* = 0.008), and hypersexuality (0.3 ± 0.7 vs. 0.0 ± 0.3, *p* = 0.006), after correction for disease severity (see Fig. 1, *p*-values corrected for multiple comparisons).

Women reached lower scores, corrected for disease stage, in Trail Making Test parts A and B (104.5 ± 125.1 vs. 150.4 ± 156.0; 245.7 ± 153.9 vs. 311.5 ± 151.7, both *p* = 0.003), semantic fluency (25.1 ± 12.6 vs. 22.0 ± 12.0, *p* = 0.03), Short Story Recall Test (9.0 ± 4.7 vs. 6.8 ± 4.8, *p* = 0.003), and Rey Complex Figure, copy (25.8 ± 14.6 vs. 21.7 ± 10.9, *p* = 0.005) (see Table 2, *p*-values corrected for multiple comparisons).

### Biological markers and survival

**Fig. 2:**
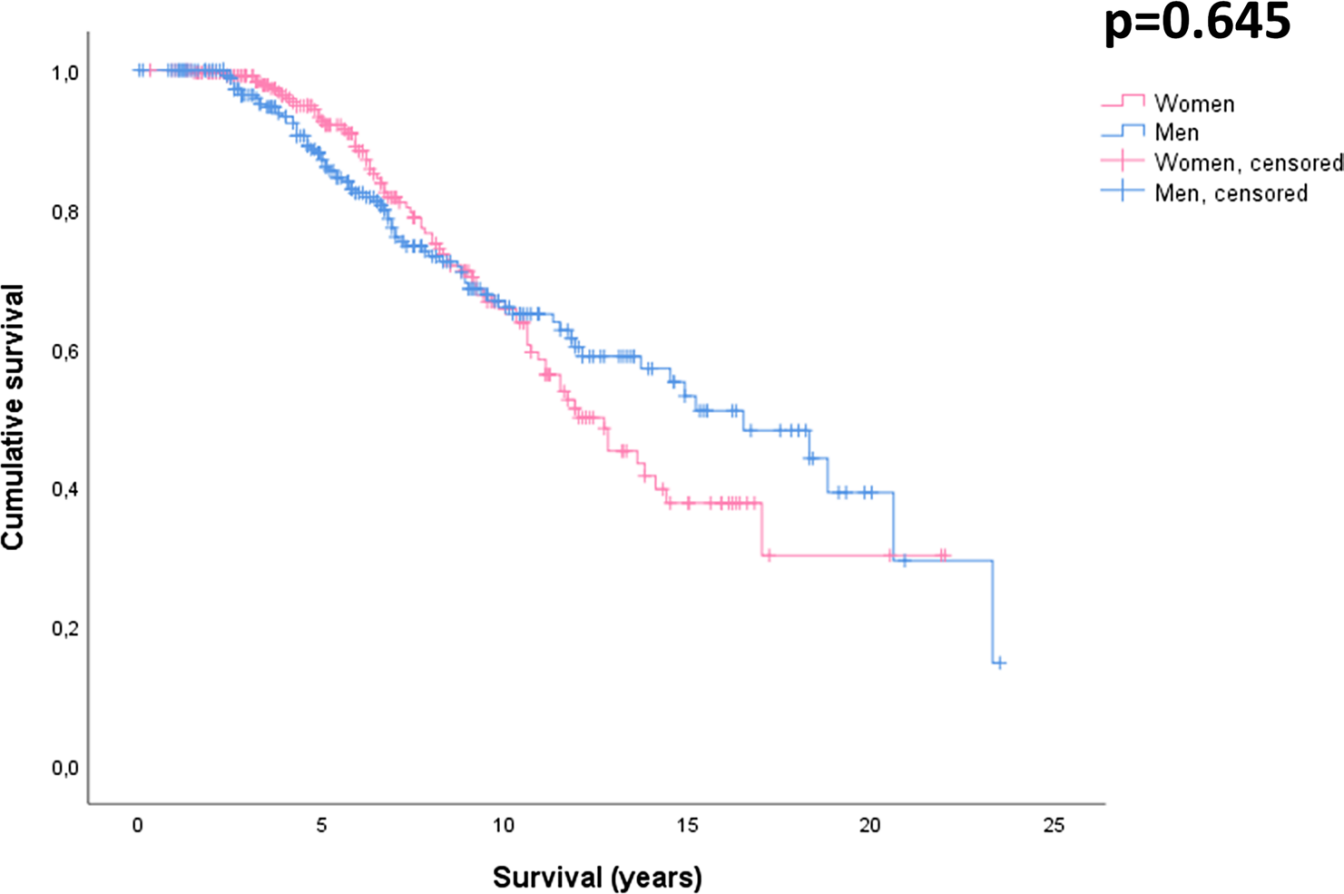
Survival in FTD patients by sex. Kaplan–Meier survival curves for men and women with FTD

NfL concentration was not different between men and women (41.2 ± 30.6 pg/ml vs 47.7 ± 30.5 pg/ml, *p* = 0.167) and across FTD subtypes (bvFTD = 43.5 ± 42.9 pg/ml, avPPA = 45.5 ± 34.5 pg/ml, svPPA = 31.8 ± 14.8 pg/ml, *p* = 0.517). The survival, assesses by Kaplan-Meyer curve, was not statistically different between men and women (*p* = 0.645) (see Fig. 2).

## Discussion

The present study demonstrated that sex influences clinical features in a wide cohort of FTD patients. Indeed, we found that bvFTD was more common in men, while women presented more frequently with PPA. The different prevalence of the two phenotypes between sex was confirmed by neuropsychological tests and assessment of behavioral disturbances. Despite a comparable general cognitive status, women performed worse in language tasks as well as in executive, visual attention, and visuospatial tests. On the opposite side, men showed a higher frequency of neurobehavioral disturbances, in particular apathy, irritability, poor judgment, aggressivity, and hypersexuality. These discrepancies were not influenced by disease stage nor by education. These observations confirm and expand the results of previous studies [15–17], and argue for a possible influence of sex in FTD as well, beyond the well-known effect in AD. The opposite prevalence of bvFTD and PPA might reflect differences in biological vulnerability between males and females.

Considering that bvFTD usually involves the right hemisphere, whereas PPA the left hemisphere, we can hypothesize the presence of an asymmetric brain vulnerability to FTD between gender. Several studies have pointed to sex-related differences in brain asymmetry in physiological conditions [27–29], some indicating that males had greater rightward lateralization, while females had greater leftward lateralization [30]. Sex differences in brain organization are thought to underlie sex differences in motor and visuospatial skills, linguistic performance, and vulnerability to deficits following stroke and other focal lesions [31] as well as pathologies disrupting brain asymmetry, namely autism, and schizophrenia [32, 33]. Thus, this asymmetric susceptibility might also explain why men develop more frequently bvFTD and women PPA.

Neuropsychological profiles, especially language impairment, and behavioral disturbances found in our FTD cohort are in line to what was observed also in AD [34–36], thus suggesting that sex per se influences the clinical presentation regardless of the specific neurodegenerative disease. This hypothesis is further supported by the observation that also in patients affected by Parkinson’s disease, men present more frequently behavioral problems [37]. Exploring the biological factors, such as hormonal influence, may shed some light on FTD pathogenesis.

According to literature data, the FTD prevalence was found similar in men and women [11], although age at onset was lower in men than in women, independently from clinical phenotype or education. This result might be a sample bias, as studies in which only bvFTD were included observed comparable disease onset between men and women [17]. In addition, women might be more attentive than men to notice behavioral and cognitive symptoms of the spouse, thus leading to an earlier diagnosis of FTD in men. On the contrary, subtle language deficits with preserved functioning in daily life activities, without any behavioral disturbances in women may be underscored by male spouses and at the same time more socially acceptable, thus conditioning a later recognition of the disease.

Finally, we found that survival and NfL concentration were comparable between men and women, thus suggesting an equal aggressiveness, as already supported by previous studies [11, 38]. However, considering the longer life expectancy for women in the general population [39], this result could be interpreted as indirect evidence of more aggressive progression and worse prognosis in females affected by FTD.

We acknowledge that the present study entails some limits. Our results relied on a large clinical cohort; however, we are aware that they should be confirmed by international epidemiological studies and pathological confirmation would be needed. Moreover, we did not consider other potential factors influencing different clinical phenotypes between sex. Finally, clinical observations should be correlated with investigations exploring structural and functional neuroimaging.

In conclusion, we have reported relevant differences in FTD patients depending on sex, in particular concerning clinical presentation. This could suggest that sex is implicated in FTD pathogenesis; defining the sex-related mechanisms involved would be of crucial significance to understand the pathophysiology of the disease and to define tailored clinical approaches.

## Supplementary Information

Below is the link to the electronic supplementary material.

Supplementary file1 (DOCX 56 KB)

## References

[1] Eliot L (2011). The trouble with sex differences. Neuron.

[2] Ruigrok ANV, Salimi-Khorshidi G, Lai MC (2014). A meta-analysis of sex differences in human brain structure. Neurosci Biobehav Rev.

[3] Lotze M, Domin M, Gerlach FH, et al (2019) Novel findings from 2,838 Adult Brains on Sex Differences in Gray Matter Brain Volume. Sci Rep 9. 10.1038/S41598-018-38239-2 10.1038/s41598-018-38239-2 PMC6368548 30737437

[4] Oveisgharan S, Arvanitakis Z, Yu L (2018). Sex differences in Alzheimer’s disease and common neuropathologies of aging. Acta Neuropathol.

[5] Mauvais-Jarvis F, Bairey Merz N, Barnes PJ (2020). Sex and gender: modifiers of health, disease, and medicine. Lancet (London, England).

[6] Ferretti MT, Iulita MF, Cavedo E (2018). Sex differences in Alzheimer disease — The gateway to precision medicine. Nat Rev Neurol.

[7] Nebel RA, Aggarwal NT, Barnes LL (2018). Understanding the impact of sex and gender in Alzheimer’s disease: A call to action. Alzheimers Dement.

[8] Snyder HM, Asthana S, Bain L (2016). Sex biology contributions to vulnerability to Alzheimer’s disease: A think tank convened by the Women’s Alzheimer’s Research Initiative. Alzheimers Dement.

[9] Altmann A, Tian L, Henderson VW, Greicius MD (2014). Sex modifies the APOE-related risk of developing Alzheimer disease. Ann Neurol.

[10] Farrer LA, Cupples LA, Haines JL (1997). Effects of age, sex, and ethnicity on the association between apolipoprotein E genotype and Alzheimer disease. A meta-analysis. APOE and Alzheimer Disease Meta Analysis Consortium. JAMA.

[11] Onyike CU, Diehl-Schmid J (2013). The epidemiology of frontotemporal dementia. Int Rev Psychiatry.

[12] Rascovsky K, Hodges JR, Knopman D (2011). Sensitivity of revised diagnostic criteria for the behavioural variant of frontotemporal dementia. Brain.

[13] Gorno-Tempini ML, Hillis AE, Weintraub S (2011). Classification of primary progressive aphasia and its variants. Neurology.

[14] Borroni B, Padovani A (2013). Dementia: a new algorithm for molecular diagnostics in FTLD. Nat Rev Neurol.

[15] Johnson JK, Diehl J, Mendez MF (2005). Frontotemporal lobar degeneration: demographic characteristics of 353 patients. Arch Neurol.

[16] Rogalski E, Rademaker A, Weintraub S (2007). Primary progressive aphasia: relationship between gender and severity of language impairment. Cogn Behav Neurol.

[17] Illán-Gala I, Casaletto KB, Borrego-Écija S (2021). Sex differences in the behavioral variant of frontotemporal dementia: A new window to executive and behavioral reserve. Alzheimers Dement.

[18] Curtis AF, Masellis M, Hsiung GYR (2017). Sex differences in the prevalence of genetic mutations in FTD and ALS. Neurology.

[19] de la Sablonnière JL, Tastevin M, Lavoie M, Laforce R (2021) Longitudinal changes in cognition, behaviours, and functional abilities in the three main variants of primary progressive aphasia: A literature review. Brain Sci 11. 10.3390/BRAINSCI11091209 10.3390/brainsci11091209 PMC8466869 34573229

[20] Fostinelli S, Ciani M, Zanardini R (2018). The heritability of frontotemporal lobar degeneration: Validation of pedigree classification criteria in a Northern Italy Cohort. J Alzheimers Dis.

[21] Borroni B, Benussi A, Archetti S (2015). Csf p-tau181/tau ratio as biomarker for TDP pathology in frontotemporal dementia. Amyotroph Lateral Scler Frontotemporal Degener.

[22] [Italian standardization and classification of Neuropsychological tests. The Italian Group on the Neuropsychological Study of Aging] - PubMed. https://pubmed.ncbi.nlm.nih.gov/3330072/. Accessed 1 Mar 2022

[23] Katz S, Ford AB, Moskowitz RW (1963). Studies of illness in the aged. The index of ADL: A standardized measure of biological and psychosocial function. JAMA.

[24] Alberici A, Geroldi C, Cotelli M (2007). The Frontal Behavioural Inventory (Italian version) differentiates frontotemporal lobar degeneration variants from Alzheimer’s disease. Neurol Sci.

[25] Kertesz A, Davidson W, Fox H (1997). Frontal behavioral inventory: diagnostic criteria for frontal lobe dementia. Can J Neurol Sci.

[26] Miyagawa T, Brushaber D, Syrjanen J (2020). Utility of the global CDR ® plus NACC FTLD rating and development of scoring rules: Data from the ARTFL/LEFFTDS Consortium. Alzheimers Dement.

[27] Galaburda AM, LeMay M, Kemper TL, Geschwind N (1978). Right-left asymmetrics in the brain. Science.

[28] Toga AW, Thompson PM (2003). Mapping brain asymmetry. Nat Rev Neurosci.

[29] Hirnstein M, Hugdahl K, Hausmann M (2019). Cognitive sex differences and hemispheric asymmetry: A critical review of 40 years of research. Laterality.

[30] Tomasi D, Volkow ND (2012). Laterality patterns of brain functional connectivity: Gender effects. Cereb Cortex.

[31] Kimura D (1999) Sex and cognition. 217

[32] Baron-Cohen S, Knickmeyer RC, Belmonte MK (2005). Sex differences in the brain: implications for explaining autism. Science.

[33] Narr KL, Thompson PM, Sharma T (2001). Three-dimensional mapping of gyral shape and cortical surface asymmetries in schizophrenia: Gender effects. Am J Psychiatry.

[34] Ott BR, Tate CA, Gordon NM, Heindel WC (1996). Gender differences in the behavioral manifestations of Alzheimer’s disease. J Am Geriatr Soc.

[35] Mega MS, Cummings JL, Fiorello T, Gornbein J (1996). The spectrum of behavioral changes in Alzheimer’s disease. Neurology.

[36] Pusswald G, Lehrner J, Hagmann M (2015). Gender-specific differences in cognitive profiles of patients with Alzheimer’s disease: Results of the Prospective Dementia Registry Austria (PRODEM-Austria). J Alzheimers Dis.

[37] Fernandez HH, Lapane KL, Ott BR, Friedman JH (2000). Gender differences in the frequency and treatment of behavior problems in Parkinson’s disease. Mov Disord.

[38] Roberson ED, Hesse JH, Rose KD (2005). Frontotemporal dementia progresses to death faster than Alzheimer disease. Neurology.

[39] Ferretti MT, Martinkova J, Biskup E (2020). Sex and gender differences in Alzheimer’s disease: current challenges and implications for clinical practice: Position paper of the Dementia and Cognitive Disorders Panel of the European Academy of Neurology. Eur J Neurol.

