# Supplementary material for: Sex influences clinical phenotype in frontotemporal dementia
Source: Neurol Sci. 2022 Jun 8;43(9):5281–7. doi: 10.1007/s10072-022-06185-7 (PMC9385756; doi:10.1007/s10072-022-06185-7)
Supplement: Supplementary file 1 — Supplementary file1 (DOCX 56 KB) [file 10072_2022_6185_MOESM1_ESM.docx]

**Table 1. Behavioral characteristics of FTD patients.**

|  | **All** | **Males** | **Females** | ***p-*values** |
| --- | --- | --- | --- | --- |
| **FBI A** |  |  |  |  |
| Apathy | 1.5±1.1 | 1.6±1.1 | 1.3±1.1 | **0.02** |
| Aspontaneity | 1.3±1.2 | 1.4±1.2 | 1.2±1.2 | 0.234 |
| Emotional Flatness | 1.0±1.1 | 1.2±1.2 | 0.9±1.1 | 0.18 |
| Inflexibility | 1.0±1.1 | 1.1±1.2 | 0.9±1.1 | 0.18 |
| Personal Neglect | 1.0±1.1 | 1.1±1.2 | 0.8±1.1 | 0.656 |
| Disorganization | 1.3±1.1 | 1.2±1.1 | 1.4±1.1 | 0.234 |
| Inattention | 1.2±1.1 | 1.2±1.1 | 1.2±1.1 | 0.982 |
| Loss of insight | 1.2±1.2 | 1.3±1.2 | 1.0±1.1 | 0.114 |
| Logopenia | 1.2±1.2 | 1.2±1.2 | 1.2±1.2 | 0.982 |
| Semantic deficit | 0.5±1.0 | 0.5±0.9 | 0.6±1.0 | 0.278 |
| Aphasia and Verbal apraxia | 1.0±1.1 | 1.0±1.1 | 1.0±1.2 | 0.982 |
| Alien hand and/or apraxia | 1.0±1.1 | 0.9±1.1 | 0.2±0.6 | 0.982 |
| **FBI B** |  |  |  |  |
| Perseveration | 0.8±1.1 | 0.9±1.1 | 0.7±1.1 | 0.055 |
| Irritability | 0.9±1.0 | 1.1±1.0 | 0.8±1.0 | **0.006** |
| Excessive jocularity | 0.3±0.7 | 0.4±0.7 | 0.3±0.7 | 0.485 |
| Poor judgment | 0.8±1.1 | 0.9±1.2 | 0.7±1.0 | **0.033** |
| Hoarding | 0.6±0.9 | 0.3±0.8 | 0.2±0.7 | 0.635 |
| Inappropriateness | 0.6±0.9 | 0.7±1.0 | 0.6±0.9 | 0.055 |
| Restlessness/Roaming | 0.3±0.8 | 0.4±0.8 | 0.3±0.8 | 0.866 |
| Aggressivity | 0.4±0.8 | 0.6±0.9 | 0.3±0.7 | **0.008** |
| Hyperorality/food fats | 0.7±1.1 | 0.8±1.1 | 0.5±0.9 | 0.055 |
| Hypersexuality | 0.2±0.5 | 0.3±0.7 | 0.0±0.3 | **0.006** |
| Utilization Behaviour | 0.2±0.6 | 0.2±0.6 | 0.2±0.6 | 0.866 |
| Incontinence | 0.1±1.1 | 0.4±1.3 | 0.3±0.7 | 0.769 |

Results are expressed as mean ± standard deviation.

FBI = Frontal Behavior Inventory.

*p-*values for one-way ANCOVA are expressed after adjusting for CDR plus NACC FTLD - SOB. All results are corrected for multiple comparisons (False Discovery Rate).
